# A Qualitative Study To Understand Parental, Health Care Provider and WIC Nutritionist Perspectives on Early Childhood Beverage Choices for WIC-enrolled Families in a Southeastern US Health System

**DOI:** 10.1007/s10995-025-04075-w

**Published:** 2025-04-01

**Authors:** Sophia Ali, Sydney-Evelyn Gibbs, Kimberly Wiseman, Jamie Zoellner, Kimberly Montez, Alysha Taxter, Mallory Suarez, Leah Hindel, Kristina H. Lewis

**Affiliations:** 1 Division of Public Health Sciences, Wake Forest University Health Sciences, Winston Salem, NC USA; 2 Department of Public Health Sciences, University of Virginia, Charlottesville, VA USA; 3 Department of Pediatrics, Wake Forest School of Medicine, Winston Salem, NC USA; 4 Department of Rheumatology, Nationwide Children’s Hospital, Columbus, OH USA

**Keywords:** Sugar-sweetened beverage, Fruit juice, Nutrition, WIC, Low income, Obesity

## Abstract

**Objectives:**

Nutritionists for the Special Supplemental Nutrition Program for Women, Infants, and Children (WIC), along with healthcare providers, can influence beverage choices for young children in WIC-enrolled families. Coordination of messaging about beverages and between-provider communication may be important for facilitating behavior change.

**Methods:**

In preparation for a planned intervention, during Spring through Fall of 2021, we conducted a qualitative study to understand perceptions around family beverage choice discussions across three groups: parents of WIC-enrolled children, WIC nutritionists and practicing clinicians. Semi-structured interviews were conducted with 25 individuals, with sample size determined a priori. Thematic content analysis was used to summarize transcribed data.

**Results:**

There was broad agreement that children should not consume sugar-sweetened beverages (SSB), but disagreement on tap water safety. Both clinicians and WIC nutritionists reported educating families about healthy drink choices, and parents recalled similar messages during these conversations. Parents were very supportive of fruit juice as part of the WIC package, with near universal opposition among clinicians. Many parents expressed concerns about tap water. Both provider groups perceived difficulty in reaching out to each other, and felt more communication would be helpful.

**Conclusions for Practice:**

Our findings highlight a need for more regular communication between WIC nutritionists and children’s healthcare providers within our system, and more consistent parental education on juice and tap water safety. Future studies could evaluate whether these types of communication gaps and perceptions are regional or more widespread across the US.

## Significance

Prior studies explored perceptions of WIC-enrolled families regarding beverage choice. Our study builds on this by interviewing WIC nutritionists and clinicians to better inform consistent family counseling around beverage choices. Our findings showed broad agreement across groups that children should not consume SSB, but parents expressed concerns about tap water. Parents and WIC nutritionists were supportive of fruit juice being part of WIC package, while pediatric clinicians were almost universally opposed to this. Both provider groups reported difficulty in communicating with each other about patients, but felt it would be helpful if there were a way to efficiently do so.

## Introduction

Excess sugar consumption has a well-documented association with childhood obesity and other health outcomes (Bleich & Vercammen, 2018; Malik & Hu, 2022), with sugar-sweetened beverages (SSB) remaining a leading source of added sugars for American children (Bailey et al., 2018; Bleich & Vercammen, 2018). Consumption of SSB is greater among children from low-income households, contributing to disparities in obesity and other health outcomes (Bably et al., 2021; Charvet & Huffman, 2019). Although 100% fruit juice is not a SSB, it is an important contributor to total sugars consumed by young children, and may be associated with adverse health consequences or poorer diet quality if consumed early or in excess of recommended amounts (Nguyen et al., 2024; Shefferly et al., 2015; Sonneville et al., 2015; Thompson et al., 2021).

The Special Supplemental Nutrition Program for Women, Infants, and Children (WIC) is a federally-subsidized program offered to low-income pregnant and postpartum women, infants, and children up to 5 years old (Anderson et al., 2022). WIC provides supplemental foods, nutrition education, and counseling and referrals to healthcare and social services (US Department of Health and Human Services & Agriculture, 2015). Given the patient population it serves, the WIC program is ideally situated to mitigate disparities in nutritional outcomes (Charvet & Huffman, 2019), including discouraging the provision of SSB, and promoting guideline-recommended levels of fruit juice intake, namely - no fruit juice before age 1 and limiting to 4 ounces per day for toddlers(Heyman, Abrams, Section On Gastroenterology, Nutrition, & Committee On, 2017; Lott, Callahan, Welker Duffy, Story, & Daniels, 2019). Similarly, pediatricians are also positioned to provide regular education and support for healthy beverage choice in childhood and are encouraged to screen for SSB and fruit juice consumption as part of a comprehensive approach to obesity prevention (Krebs et al., 2007).

Little is known about whether these 2 groups of providers (WIC nutritionists and pediatricians) are communicating consistent, guideline-driven messages on child beverage choice to WIC participants. To better understand this issue and inform development of a planned beverage choice intervention for WIC-enrolled children, we conducted interviews with parents of WIC-enrolled children, WIC nutritionists and pediatricians.

## Methods

### Study Design & Setting

This was a qualitative study using semi-structured interviews to inform the development of a planned health-system embedded intervention to promote healthy patterns of beverage consumption among WIC-eligible or enrolled infants and toddlers. The study took place within Atrium Health Wake Forest Baptist (AHWFB), a large academic healthcare system in the Piedmont region of North Carolina. The AHWFB Institutional Review Board approved the research protocol and consent process.

### Qualitative and Patient-Reported Outcomes (Q-PRO) Shared Resource

The qualitative and Patient-Reported Outcomes (Q-PRO) Shared Resource at AHWFB is a dedicated team who provide investigators with methodologic expertise for qualitative research. Q-PRO is comprised of six expert researchers trained in qualitative methods, collectively with over 40 years’ experience.

### Parents/Caregivers

The parental sample size (*n* = 15) was designated a priori based upon prior work with similar populations (McCall et al., 2024; Newman et al., 2023), and with a goal of reaching saturation with parents/caregivers (the primary target of the planned intervention). We recruited parents or caregivers of WIC-enrolled children consuming above guideline-recommended levels of SSB or fruit juice. Parents/caregivers (collectively: parents) were considered eligible if they cared for a child under 5 who was currently participating in the WIC program and consuming 2 or more servings of fruit juice or SSB a day. A list of potentially-eligible parents was identified using child electronic health record (EHR) data (Lewis et al., 2018). Potential participants were contacted by phone to assess their interest and ability to converse in English. Informed consent was obtained verbally, with consent forms mailed based on participant preference.

### Providers (WIC and Clinician)

For the clinician and WIC nutritionist interviews, a smaller sample size (*n* = 5 each) was pre-designated because the purpose of these interviews was preliminary information gathering and identifying key areas of agreement or disagreement with parental interviews. Eligibility criteria for WIC nutritionist interviews were that the participant was currently practicing within the local county WIC program. For clinician interviews, we required that an individual was in primary care practice in our health system and treating WIC-enrolled pediatric patients. Potential participants for provider interviews were identified using standardized invitation emails to employees in both groups. Interested individuals then opted-in by emailing study staff and underwent telephone-based informed consent prior to participating.

### Interviewer Training

Interviewers (author M.S. plus two study coordinators at AHWFB) were trained in technique by the Q-PRO team. Training focused on building rapport with interviewees, maintaining neutrality and avoiding leading questions, avoiding social desirability bias, and utilizing probes to elicit deeper context and understanding.

### Development of Interview Guides and Conduct of Interviews

The parental interview guide was developed first, with input from Q-PRO team members to broadly address several domains specified by the research team as critical for intervention development and implementation, including parental perceptions around family drink choices, and recall of previous interactions with a clinician or WIC provider on these topics (Table 1).

**Table 1:** Outline and example questions from interview guides

Domain Addressed	Sample Interview Questions
**Parental interview guide**	
Consumption of SSB, Fruit Juice and Water: Facilitators	“What are the top 2 or 3 reasons why you or your children might drink a sugary drink or fruit juice?”
	“What are the top 2 or 3 reasons why you or your children might drink water?”
Consumption of SSB, Fruit Juice and Water: Barriers	“What are the top 2 or 3 reasons why you or your children might not drink water?”
Education about Healthy Drink Choices	“Can you remember any time where the WIC nutritionist talked to you about what your child should be drinking?”
	“Can you remember any time where your child’s doctor talked to you about what your child should be drinking?”
Provision of Fruit Juice by the WIC Program	“What do you think about being able to purchase fruit juice with WIC benefits?”
Communication between providers	“Has the WIC nutritionist ever asked you to speak to your child’s doctor about any kind of issue?”
	“Does your child’s doctor ever ask you to speak to your WIC nutritionist?”
***WIC nutritionist interview guide***	
Education about Healthy Drink Choices	“Compared to other topics you might discuss with WIC clients, how important is family drink choice? Why do you feel this way?”
	“What additional resources or tools might help you with educating families about healthy drink choices, including drinking less sugary drinks and more water?”
	“When it comes to drink choices for children under age 5, what is the usual advice you provide to parents or caregivers?”
Communication with Providers	“Have you ever reached out to a child’s medical provider to discuss a concern related to a child’s drink intake? What was discussed?”
	“Have you ever heard directly from a child’s pediatrician about beverage choices for one of your WIC clients?”
Provision of Fruit Juice by the WIC Program	“What are your thoughts about WIC participants being about to purchase fruit juice with their WIC benefits?”
***Clinician interview guide***	
Education about Healthy Drink Choices	“Compared to other topics you might discuss with pediatric patients and families, how important is family drink choice? Why do you feel this way?”
	“What additional resources or tools might help you with educating families about healthy drink choices, including drinking less sugary drinks and more water?”
	“When it comes to drink choices for children under age 5, what is the usual advice you provide to parents or caregivers?”
Communication with WIC	“Have you ever reached out to someone in the WIC program to discuss a concern related to a child’s drink intake? What was discussed?”
	“Have you ever heard directly from the WIC program about beverage choices for one of your patients?”
Provision of Fruit Juice by the WIC Program	“What are your thoughts about WIC participants being about to purchase fruit juice with their WIC benefits?”

After parental interviews were completed, one team member (KHL) conducted a preliminary review of recordings to inform content of WIC nutritionist and clinician interview guides. As with the parental guide, provider interview guides were written collaboratively by the research team and Q-PRO. The provider interview guides were designed to address several topics, including educating parents about beverage choices, perceptions of fruit juice and WIC benefits, communication between pediatricians and WIC nutritionists, and feedback on the proposed intervention.

All interviews were conducted 1:1 with participants over the phone and field notes were taken on a standardized form. Each interview lasted between 25 and 30 min and was digitally recorded and transcribed. Transcriptions were cleaned by the Q-PRO team by reviewing the transcript against the audio to ensure accuracy of the transcription. Representative quotations were selected and cleaned utilizing the principles of representation outlined in Whitney et al. (2024)(Whitney et al., 2024). Transcript review and quotation review involved ensuring missing data was added in and mistaken data was corrected, and sanitizing of grammar, syntax, and spelling (such as changing ‘cause’ to ‘because’).

The 15 parental interviews were completed in the Spring and Summer of 2021, and the 10 provider interviews were completed in the Fall of 2021. Interview participants were compensated with a $25 gift card.

### Data Analysis

Concurrent with data collection, interviews were monitored and reviewed for emerging themes by the interviewers and the principal investigator, and it was determined that no additional interviews were needed to ensure saturation of the data. Two Q-PRO teammates conducted a thematic content analysis of transcripts to reveal patterns and overall themes in the data. First, each reviewed two transcripts from each interviewed group (parents, clinicians, and WIC nutritionists) and developed a draft codebook for each group. The study team reviewed the codebook and provided input for revisions. The revised codebook was tested by completing coding on several additional interview transcripts and further revised. All interviews were then independently coded in Atlas.ti version 9 by two Q-PRO teammates and coding was compared; any discrepancies were discussed and resolved to consensus. Once all transcripts were coded, code reports were run and summaries for each report were written. Code summaries were analyzed for patterns and themes, which are presented below. Differences between parents, clinicians, and WIC nutritionists were explored. COREQ criteria for reporting qualitative research were reviewed and, where relevant, followed in the preparation of this manuscript.

## Results

### Participant Characteristics

One hundred and eight potentially eligible parents were reached by phone for recruitment. Seventy-three declined participation or were ineligible and 20 did not respond at the time of their scheduled interview yielding a final response rate of 14%. Parental characteristics are summarized in Table 2. Among the 5 WIC nutritionists interviewed, 3 were more recently employed by WIC (< 5 years of employment) and 2 had worked for the program for 5 or more years. All 5 clinicians interviewed were pediatric physicians. Four had been in clinical practice for 10 years or more, and 1 had been in practice for 5–9 years.

**Table 2:** Selected demographic and other measured characteristics of 15 parents/caregivers participating in interviews

Characteristic	*N* (%) or Mean (SD)
Age (years)	36.1 (11.7)
Self-Identified Race or Ethnicity	
Non-Hispanic Black	10 (67%)
Hispanic	1 (7%)
Other race or Ethnicity	4 (27%)
Marital Status	
Married	8 (53%)
Single	7 (47%)
Relationship to Child	
Mother	11 (73%)
Other (e.g. other family member, foster care provider)	4 (27%)
Educational Level	
High School or Less	7 (46%)
Some College or Greater	8 (54%)
Reported Duration of WIC Enrollment Prior to Interview	
More than 5 years	4 (27%)
2–5 Years	6 (40%)
Less than 2 years	5 (33%)

### Overview of Topics and Themes Identified across Interviews

Themes emerged surrounding five main topics: “Consumption of SSB and fruit juice”, “Consumption of Water”, “Education about Healthy Drink Choices”, “Provision of Fruit Juice by the WIC Program”, and “Communication between Providers”. For each topic, identified themes are presented in Table 3, with supporting details and representative quotes separated by interviewed group to facilitate comparison of responses.

**Table 3:** Topics, themes and representative quotes from Semi-Structured interviews

Topic: Theme(s)	Representative Quotes^a^– Grouped by Agreement			
**1. Consumption of Sugar-Sweetened Beverages and Fruit Juice:** SSB should be avoided in children and fruit juice minimized but this is difficult because of sweet taste preference and commercial influences	**Parental and Provider Perspectives** *Parent 08*: “I know my little one likes to drink it because of the sugar…it’s sweet. I buy the reduced sugar drinks. When I buy those, he does not drink it as much as the one with the regular sugar in it…Fruit juice more so because it’s healthier. Sugary drink, just mostly for the taste.” *Parent 13*: “The main reason is because we could be at an outing, a birthday party, or something, and that’s what’s provided as the drink of choice. We can be out somewhere and probably just needing to grab something from the store really quickly, and it’s a child’s choice because it’s probably the cartoon character on the bottle or the color of the juice, so giving in at the moment.” *Parent 05*: “One of the reasons is probably because they don’t have a taste for water at that moment and want something sweet.” and “I just feel that kids shouldn’t really drink soda as much because it does affect their health in the long run.” *WIC Nutritionist 01*: “Honestly, I think it’s a lot of just commercials and a lot of things that they see on TV. Advertisements.…I mean, there’s a lot of advertisement out there that leads parents to make these decisions I would say.” *Clinician 01*: “Sometimes other family members have influence and change what parents do so like, grandparents, uncles, aunts, other extended family. Of course, parents are going to listen to what their parents recommend and what their siblings recommend.”			
**2. Consumption of water**: Parents do not perceive replacement of SSBs with tap water as safe	**Parental Perspectives** *Parent 03*: “*Especially where I live*,* I don’t think it’s safe. I do not drink the water out of my faucet. I do not let my daughter drink the water out of my faucet because it has the chlorine in it and you can taste it*.” *Parent 12*: *“We don’t drink the tap water because of the– my tap water has a whole lot of chlorine in it. You can smell it.”*		**Provider Perspectives** *WIC Nutritionist 05*: “*We are lucky enough to live in an area where we do have safe drinking water*” *Clinician 02*: “*From what I understand*,* there’s not been issues with lead [in local tap water] such as*,* of course*,* in Flint*,* Michigan*”	
**3. Education about Healthy Drink Choices**: Providers report frequent discussions with parents and parents report receiving consistent messages.	**Parental and Provider Perspectives** *Parent 03*: “*For my daughter*,* I definitely have because at one point she was—once she got old enough to start drinking juices instead of just nothing but milk*,* I was giving her a lot of sugary stuff. She was drinking Kool-Aid. She was drinking sweet tea. After that*,* when I talked to the nutritionist and she was like*,* “Hey*,* you know*,* she should be having this much water a day. These are the things that she shouldn’t really be drinking a lot of. These are the things that she should be*,*” yes*,* I started giving her water more. I started changing up her drinks. I started checking the sugars on the back. Yeah*,* it did change after I talked to her.*” *WIC Nutritionist 03*: “*We’re constantly talking to them about drinking sweet drinks and how important it is that they don’t—if the child is overweight*,* if the child—you know*,* the chances of getting cavities with it. Then the chances are if the child drinks a whole lot of sweet drinks*,* they will not eat their meals. There’s so many things that can interfere with them drinking those sweet drinks like that*” *Clinician 03*: “*A bulk of the time that I spend—because you know when you’re talking about sweetened beverage intake*,* sodas*,* or like Snapple or whatever*,* that is a no brainer for a lot of families. They do understand that sugar is not good*,* but for younger kids*,* a lot of families think that 100% fruit juice is as good as eating fruits or is actually healthy. I think that’s where I spend a lot of time trying to explain to them that it is not*” *Clinician 04*: *“I think that families have a lot of misinformation*,* thinking*,* “oh*,* this comes from*,* quote*,* fruit*,* so it’s healthy*,*” but don’t understand all the additive sugar in most of the commercially available kids’ drinks*,* so I think it’s extremely important to improve education.”*			
**4. Provision of Fruit Juice by the WIC program**: Strong parental support, strong clinician opposition, with WIC holding a middle ground stance	**Parental Perspectives** *Parent 08*: “*Fruit juice? I think it’s a great idea. I mean*,* I feel like it’s a healthier choice than the sugary drinks*,* so definitely a better option*.” *Parent 03*: *“Every month. I get my WIC every month. That’s usually the first thing that I get off the WIC would be the juice and the eggs. Because my daughter goes through juice so much*,* so yeah*,* as soon as my benefits hit every month*,* that’s the first thing that I go do with it is get the juice.”*	**WIC Nutritionist Perspective** *WIC 02*: “*Well*,* WIC is based on the guidelines of USDA*,* and the recommendation we have in place for the amount of juice that’s provided*,* we’re meeting the guidelines*.”		**Clinician Perspective** *Clinician 04*: “*I think if they don’t provide financial support for these drinks*,* then the families will hopefully not buy them*,* and so if we’re recommending they choose alternatives like water*,* then I don’t think we should be giving them vouchers to purchase these drinks*.”
**5. Communication between providers**: Both provider groups perceived difficulty in reaching out to the other, but felt it would be helpful and desired a tool to facilitate communication	N/A	**WIC Nutritionist Perspective** *WIC 01*: “*I’ve called the doctor’s office. A lot of times I don’t get ahold of the actual doctor. Sometimes if it’s something that the—well*,* usually what happens is the receptionist or the nurse writes a note for the doctor and either the doctor goes ahead and fixes it and sends us back a prescription or the doctor actually calls me.*”		**Clinician Perspectives** *Clinician 03*: “*I think it’s access again because you have to call somebody*,* wait on the line*,* find someone is much harder.*” *Clinician 02*: “*Part of it*,* to be honest with you*,* is probably more because I wouldn’t even know how to reach a WIC nutritionist”*
*a– Please note that*,* as described in the Methods section*,* some quotations have been modified slightly compared to the transcript to remove un-necessary conjunctions and other colloquializations. This method of treatment for all quotations was employed to ensure that participant viewpoints were presented without risk of introducing bias or value judgement about the participant point of view based on style of speech.*				

### Topic 1: Consumption of SSB and Fruit Juice

*Theme: SSB should be avoided in children and fruit juice minimized but this is difficult because of sweet taste preference and commercial influences (*Table 3*).*

There was consensus among parents that children consumed either SSB or fruit juice due to craving sweets, broad availability, and convenience. When asked about limiting these drinks, the main reasons parents provided were concerns about a “sugar rush” and agitation/hyperactivity as well as the long-term health consequences of sugar. The main limitation tactics reported by parents were diluting a child’s drink with water or replacing it with flavored water.

WIC nutritionists noted the high prevalence of advertising and marketing of drinks to children as something that makes it harder for families to make healthy choices. For child health, WIC nutritionists highlighted how sugary drinks (either SSB or excessive fruit juice) could affect children’s eating patterns, dental health and shorter-term health outcomes like weight gain. WIC nutritionists described the guideline recommendation to allow 4–6 ounces of 100% fruit juice per day for children and were generally in alignment with this stance.

All interviewed clinicians expressed negative feelings about sweet drinks, including both SSB and fruit juice. One clinician indicated that children should not have any fruit juices, despite guideline recommendations allowing for small amounts. Another clinician stated they “absolutely hate juice” and called it “the poison”. Clinicians were focused on the effect of both beverage types (SSB and fruit juice) on child weight gain and related health problems. One clinician specifically mentioned an increasing frequency of children with fatty liver disease due to consumption of processed foods, and high triglyceride levels due to corn syrup consumption. When asked about facilitators of unhealthy drink choice in families, one clinician noted that family members perceived as senior (e.g., grandparents, aunts) can negatively influence parents’ choices.

### Topic 2: Consumption of Water

#### Theme: Parents Do not Perceive Replacement of SSBs with Tap Water as Safe (see Table 3)

Although all three interviewed groups agreed about limiting SSB for infants and toddlers, they disagreed on replacement of SSB with tap water. Most parents reported health benefits of their child drinking water, including fluoride in tap water for dental health and the importance of maintaining hydration. Fourteen (93%) parents indicated a distrust of tap water and sentiment that it was unsafe, with reasons including brown color, and the smell and taste of chlorine. No parent reported their family drinking exclusively tap water, with eleven (73%) reporting only drinking bottled water. Parents didn’t report hearing that tap water was unsafe from any source in particular, but some attributed the belief to their experiences growing up.

Among interviewed WIC nutritionists, all reported a perception that local tap water was a safe option, but two indicated specifically that for infants, purified or filtered water should be used. All interviewed clinicians reported a perception that tap water was a safe alternative beverage to SSB or fruit juice, though one clinician clarified that well water was not recommended for infants.

### Topic 3: Education about Healthy Drink Choices

#### Theme: Providers Report Frequent Discussions with Parents about Beverage Choices, and Parents Report Receiving Consistent Messages (see Table 3)

Providers placed priority on educating families about healthy drink choices. Despite some perceived differences between providers (e.g. a perception by pediatricians that WIC may counsel differently than a clinician), parents recalled similar messages from these conversations.

Most parents reported discussing beverages with their child’s WIC nutritionist and clinician. The main message parents took away from discussions with providers was that children should drink more water and avoid all sugary drinks. Most parents reported that they had reduced the amount of sugar in their child’s diet based on the recommendation of either a WIC nutritionist or clinician. Nine parents (60%) reported that a WIC nutritionist advised them on the appropriate amount of fruit juice for their child, which most parents remembered as 1–2 servings/day. Parents reported that both WIC nutritionists and clinicians educated them about 100% fruit juice and its sugar content.

WIC nutritionists spoke extensively about their role in educating parents, including discussing health effects of SSB and fruit juice, particularly risk of childhood obesity and dental caries. WIC nutritionists noted frequently correcting parental misconceptions, especially around 100% fruit juice, however, none advised parents to completely avoid fruit juice for their children. Instead, they reported counseling on strategies such as staying within the recommended serving size for fruit juice, diluting juice, and finding lower-sugar alternatives such as carbonated/flavored water. Regarding education around water consumption, WIC nutritionists reported that they did not tend to initiate this conversation but did advise on the safety and benefits of tap water if asked.

All interviewed clinicians reported discussing beverage choice at well-child visits if they had concerns (e.g., based on child BMI, dental health), or to correct parental misconceptions about specific drinks like fruit juice. Interviewed clinicians tended to advise little or no fruit juice in these conversations. Three clinicians reported discussing the health effects of SSB and fruit juice with parents, citing weight gain and associated health issues including diabetes. The primary tactic suggested to reduce SSB and fruit juice consumption was for parents to completely avoid having either beverage in the house. Like WIC nutritionists, clinicians said they did not initiate conversations about tap water but would advise on its safety and benefits if asked.

### Topic 4: Provision of Fruit Juice by the WIC Program

#### Theme: Strong Parental Support, Strong Clinician Opposition, with WIC Holding a Middle Ground Stance (see Table 3)

Parents were universally positive about the fact that WIC benefits include a provision for fruit juice purchase using electronic benefit transfer (EBT) cards and agreed that the current amount of fruit juice vouchers was enough. For some parents, juice was among the first purchases they make when the EBT voucher arrives each month. All interviewed parents indicated it was good that fruit juice was being provided by WIC benefits because otherwise fruit juice was expensive.

Most WIC nutritionists were supportive of the fact that WIC provides fruit juice vouchers. Two nutritionists pointed out that the amount of 100% fruit juice provided each month meets the daily guidelines for pediatric consumption. Two nutritionists mentioned that they have had parents suggest that WIC should provide more fruit instead of fruit juice.

All five clinicians had negative opinions about fruit juice vouchers being provided by WIC, as they felt it communicated mixed messaging to families and led to excessive fruit juice intake.

### Topic 5: Communication between Providers

#### Theme: both Provider Groups Perceived Difficulty in Reaching Out To the Other, but Felt It Would Be Helpful and Desired a Tool To Facilitate Communication (see Table 3)

Both interviewed provider groups perceived difficulty in reaching out to each other, but felt it would be helpful if there was more communication. Only one WIC nutritionist reported that a clinician had reached out to them in the past (about fruit juice). In contrast, all WIC nutritionists reported having reached out to a clinician. They reported this communication was primarily focused on discussions of milk, formula, and other nutritional supplements in relation to a child’s weight. No WIC nutritionist reported reaching out to a clinician about SSB. WIC nutritionists reported frequently reaching out to clinicians by phone, although they noted that they could not be connected directly to the clinician. Almost all WIC nutritionists indicated a desire for a better system of communication with clinicians.

All but one clinician reported having reached out to WIC nutritionists and stated that their outreach had been focused on discussions of milk, formula, and other nutritional supplements in relation to a child’s weight. One clinician reported perceiving consensus between clinicians and WIC on the topic of family beverage choices, while two others reported perceiving a lack of consensus. Almost all clinicians indicated a desire for a better system to communicate with WIC.

## Discussion

In this qualitative study about child beverage choices among WIC-enrolled families, pediatric clinicians conveyed a stricter view on SSBs and more negative views on fruit juice than WIC nutritionists and parents. Although all interviewed providers felt that tap water was an ideal substitute for SSB or fruit juice, it was viewed as unsafe by parents in our sample. Our results highlight the need for work to understand the scope of these issues beyond our system, and, locally, a need to improve communication between providers.

Although several prior studies have explored the perceptions of WIC-enrolled families regarding SSB, fruit juice, and water, our study builds on this literature by interviewing WIC nutritionists and clinicians. Consistent with other studies (Morel et al., 2019), parents in our sample described their child’s taste preference for “sweet”, and parental convenience as driving factors for child SSB and fruit juice consumption. Similar to several other studies, providers in our sample indicated that parents often perceive fruit juice or fruit-flavored drinks as a healthy option for children (McElligott et al., 2012; Moran & Roberto, 2018; Morel et al., 2019; Vercammen et al., 2018), and cited the influence of targeted marketing or advertising, another theme that has been expressed in prior studies with WIC-enrolled families (Morel et al., 2019).

WIC nutritionists and clinicians in our sample held different views on WIC’s provision of fruit juice. Interviewed clinicians worried that, by providing fruit juice, WIC could create a mixed message. They took a more rigid stance on complete avoidance of fruit juice for small children. The WIC nutritionists we interviewed adopted more of a “harm reduction” stance, that fruit juice is not as good as water, but it is a better choice than SSB.

Health behavior education from providers can positively impact nutritional and other lifestyle choices made by patients(Eckel et al., 2014; Halm & Amoako, 2008; Oberg & Frank, 2009). This idea was supported in our interviews, as parents repeatedly described making changes to their child’s SSB and fruit juice intake based on prior education from their WIC nutritionist and/or clinician. A current missed opportunity may therefore be expanding these conversations to routinely address tap water intake. Although both interviewed provider groups indicated they would talk about tap water if prompted, it was not something that they addressed with every family. Given that all interviewed parents perceived tap water was unsafe, and many were exclusively purchasing bottled water for their families, this topic could be addressed routinely during conversations in our health system about beverage choice with WIC-enrolled or eligible families. This same theme of parental concern about safety of tap water and preference for bottled water has emerged in other studies exploring barriers to water consumption in similar populations (Beck et al., 2014; Morel et al., 2019).

Lastly, both groups of providers in our study expressed a general perception of communication between WIC and the health system being a “one-way street” - although each group reported frequent outreach to the other, neither felt their outreach was routinely reciprocated. Given the potential health benefits of greater care coordination (Bailey-Davis et al., 2018), this finding highlights the need for understanding whether there is demand beyond our health system for strategies to make WIC-clinician provider communication easier and more frequent. Such conversations might prevent confusion on the part of parents and could serve to promote provider synergy on shared goals such as increasing water consumption, decreasing SSB, promoting guideline-appropriate levels of fruit juice intake, and reinforcing messages from one another.

There are several important limitations of the study. Because parents/caregivers were selected for interviews based on having young children in WIC who over-consumed either SSB or fruit juice, their views may not be transferrable to families not enrolled in WIC, or those whose children are consuming at or below guideline-recommended levels of these beverages. Also limiting transferability is the fact that all participants were selected from one health system in a distinct geographic region in the Southeastern US; thus, our findings may not be reflected among other populations. Our sample of Hispanic parents was limited, with only 1 of the 15 parents identifying as a person of Hispanic origin. This was likely related in part to our requirement for English speaking. Some parents in our sample may have found questions about child beverage choice to be a sensitive topic, thus it is possible that social desirability bias impacted their responses. Lastly, the timing of the interviews coincided with the COVID-19 pandemic, which could have resulted in lower parental response rate or altered parent or child behaviors around beverage consumption.

## Conclusion

WIC nutritionists and clinicians have frequent contact with nutritionally at-risk families, and are uniquely positioned to provide key education and counseling messages regarding beverage choices. This qualitative study revealed areas of disagreement between provider groups and WIC-enrolled parents in our health system. Additional work in similar populations could further elucidate how widespread these issues are and clarify the need for programs to promote more consistent provider counseling. Communication of consistent messages on beverage choice to WIC parents is a goal that may be more readily achieved with better care coordination and communication between the WIC program and health systems.

## Data Availability

Interview scripts are available upon request.

## References

[CR1] Anderson, C. E., O’Malley, K., Martinez, C. E., Ritchie, L. D., & Whaley, S. E. (2022). Longer family participation in WIC is associated with lower childhood Sugar-Sweetened beverage intake. *Journal of Nutrition Education and Behavior*, *54*(3), 239–248. 10.1016/j.jneb.2021.10.003 35000830 10.1016/j.jneb.2021.10.003

[CR2] Bably, M. B., Paul, R., Laditka, S. B., & Racine, E. F. (2021). Factors associated with the initiation of added sugar among Low-Income young children participating in the special supplemental nutrition program for women, infants, and children in the US. *Nutrients*, *13*(11). 10.3390/nu13113888 10.3390/nu13113888 PMC8624134 34836143

[CR4] Bailey, R. L., Fulgoni, V. L., Cowan, A. E., & Gaine, P. C. (2018). Sources of added sugars in young children, adolescents, and adults with low and high intakes of added sugars. *Nutrients*, *10*(1). 10.3390/nu10010102 10.3390/nu10010102 PMC5793330 29342109

[CR3] Bailey-Davis, L., Kling, S. M. R., Cochran, W. J., Hassink, S., Hess, L., Hosterman, F., & Savage, J., J. S (2018). Integrating and coordinating care between the women, infants, and children program and pediatricians to improve patient-centered preventive care for healthy growth. *Transl Behav Med*, *8*(6), 944–952. 10.1093/tbm/ibx046 29370433 10.1093/tbm/ibx046

[CR5] Beck, A. L., Takayama, J. I., Halpern-Felsher, B., Badiner, N., & Barker, J. C. (2014). Understanding how Latino parents choose beverages to serve to infants and toddlers. *Maternal and Child Health Journal*, *18*(6), 1308–1315. 10.1007/s10995-013-1364-0 24077961 10.1007/s10995-013-1364-0

[CR6] Bleich, S. N., & Vercammen, K. A. (2018). The negative impact of sugar-sweetened beverages on children’s health: An update of the literature. *BMC Obes*, *5*, 6. 10.1186/s40608-017-0178-9 29484192 10.1186/s40608-017-0178-9 PMC5819237

[CR7] Charvet, A. (2019). G. F. Huffman (Ed.), Beverage intake and its effect on body weight status among WIC Preschool-Age children. *J Obes* 2019 3032457 10.1155/2019/3032457. 10.1155/2019/3032457 PMC6360070 30800480

[CR8] Eckel, R. H., Jakicic, J. M., Ard, J. D., de Jesus, J. M., Houston Miller, N., & Hubbard, V. S. (2014). 2013 AHA/ACC guideline on lifestyle management to reduce cardiovascular risk: a report of the American College of Cardiology/American Heart Association Task Force on Practice Guidelines. *Journal Of The American College Of Cardiology*, *63*(25 Pt B), 2960–2984. 10.1016/j.jacc.2013.11.003. American College of Cardiology/American Heart Association Task Force on Practice. 10.1016/j.jacc.2013.11.003 24239922

[CR9] Halm, J., & Amoako, E. (2008). Physical activity recommendation for hypertension management: Does healthcare provider advice make a difference? *Ethnicity and Disease*, *18*(3), 278–282. 18785439

[CR10] Heyman, M. B., Abrams, S. A., On Gastroenterology, S., Nutrition, H., & On, C., N (2017). Fruit juice in infants, children, and adolescents: Current recommendations. *Pediatrics*, *139*(6). 10.1542/peds.2017-0967 10.1542/peds.2017-0967 28562300

[CR11] Krebs, N. F., Himes, J. H., Jacobson, D., Nicklas, T. A., Guilday, P., & Styne, D. (2007). Assessment of child and adolescent overweight and obesity. *Pediatrics*, *120*(Suppl 4), S193–228. 10.1542/peds.2007-2329D 10.1542/peds.2007-2329D 18055652

[CR12] Lewis, K. H., Skelton, J. A., Hsu, F. C., Ezouah, P., Taveras, E. M., & Block, J. P. (2018). Implementing a novel electronic health record approach to track child sugar-sweetened beverage consumption. *Prev Med Rep*, *11*, 169–175. 10.1016/j.pmedr.2018.06.007 29988772 10.1016/j.pmedr.2018.06.007 PMC6031146

[CR13] Lott, M., Callahan, E., Welker Duffy, E., Story, M., & Daniels, S. (2019). *Healthy Beverage Consumption in Early Childhood: Recommendations from Key National Health and Nutrition Organizations*. Retrieved from Durham NC: https://healthydrinkshealthykids.org/app/uploads/2019/09/HER-HealthyBeverageTechnicalReport.pdf

[CR14] Malik, V. S., & Hu, F. B. (2022). The role of sugar-sweetened beverages in the global epidemics of obesity and chronic diseases. *Nature Reviews. Endocrinology*, *18*(4), 205–218. 10.1038/s41574-021-00627-6 35064240 10.1038/s41574-021-00627-6 PMC8778490

[CR15] McCall, A., Strahley, A. E., Martin-Fernandez, K. W., Lewis, K. H., Pack, A., Ospino-Sanchez, B., & Montez, K. G. (2024). WIC staff and healthcare professional perceptions of an EHR intervention to facilitate referrals to and improve communication and coordination with WIC: A qualitative study. *J Clin Transl Sci*, *8*(1), e47. 10.1017/cts.2024.488 38510692 10.1017/cts.2024.488 PMC10951923

[CR16] McElligott, J. T., Roberts, J. R., Varadi, E. A., O’Brien, E. S., Freeland, K. D., & Basco, W. T. Jr (2012). Variation in fruit juice consumption among infants and toddlers: Associations with WIC participation. *Southern Medical Journal*, *105*(7), 364–369. 10.1097/SMJ.0b013e31825c0252 22766665 10.1097/SMJ.0b013e31825c0252

[CR17] Moran, A. J., & Roberto, C. A. (2018). Health warning labels correct parents’ misperceptions about sugary drink options. *American Journal of Preventive Medicine*, *55*(2), e19–e27. 10.1016/j.amepre.2018.04.018 29903567 10.1016/j.amepre.2018.04.018 PMC6128141

[CR18] Morel, K., Nichols, K., Nong, Y., Charles, N., Price, S., Taveras, E., & Woo Baidal, J. A. (2019). Parental and provider perceptions of Sugar-Sweetened beverage interventions in the first 1000 days: A qualitative study. *Academic Pediatric*, *19*(7), 748–755. 10.1016/j.acap.2019.01.004 10.1016/j.acap.2019.01.004 PMC6642844 30677540

[CR19] Newman, C. M., Zoellner, J., Schwartz, M. B., Pena, J., Wiseman, K. D., Skelton, J. A., & Lewis, K. H. (2023). Knowing is not doing: A qualitative study of parental views on family beverage choice. *Nutrients*, *15*(12). 10.3390/nu15122665 10.3390/nu15122665 PMC10305040 37375569

[CR20] Nguyen, M., Jarvis, S. E., Chiavaroli, L., Mejia, S. B., Zurbau, A., Khan, T. A., & Malik, V. S. (2024). Consumption of 100% fruit juice and body weight in children and adults: A systematic review and Meta-Analysis. *JAMA Pediatr*, *178*(3), 237–246. 10.1001/jamapediatrics.2023.6124 38227336 10.1001/jamapediatrics.2023.6124 PMC10792499

[CR21] Oberg, E. B., & Frank, E. (2009). Physicians’ health practices strongly influence patient health practices. *J R Coll Physicians Edinb*, *39*(4), 290–291. 10.4997/JRCPE.2009.422 21152462 10.4997/JRCPE.2009.422 PMC3058599

[CR22] Shefferly, A., Scharf, R. J., & DeBoer, M. D. (2015). Longitudinal evaluation of 100% fruit juice consumption on BMI status in 2-5-year-old children. *Pediatr Obes*. 10.1111/ijpo.12048 26110996 10.1111/ijpo.12048 PMC4734899

[CR23] Sonneville, K. R., Long, M. W., Rifas-Shiman, S. L., Kleinman, K., Gillman, M. W., & Taveras, E. M. (2015). Juice and water intake in infancy and later beverage intake and adiposity: Could juice be a gateway drink? *Obesity (Silver Spring)*, *23*(1), 170–176. 10.1002/oby.20927 25328160 10.1002/oby.20927 PMC4276519

[CR24] Thompson, I. J. B., Ritchie, L. D., Bradshaw, P. T., Mujahid, M. S., & Au, L. E. (2021). Earlier introduction to Sugar-Sweetened beverages associated with lower diet quality among WIC children at age 3 years. *Journal of Nutrition Education and Behavior*, *53*(11), 912–920. 10.1016/j.jneb.2021.04.468 34229969 10.1016/j.jneb.2021.04.468

[CR25] US Department of Health and Human Services, &, & Agriculture, U. D. (2015). o. *2015–2020 Dietary Guidelines for Americans*. Retrieved from http://www.health.gov/DietaryGuidelines

[CR26] Vercammen, K. A., Moran, A. J., Zatz, L. Y., & Rimm, E. B. (2018). 100% juice, fruit, and vegetable intake among children in the special supplemental nutrition program for women, infants, and children and nonparticipants. *American Journal of Preventive Medicine*, *55*(1), e11–e18. 10.1016/j.amepre.2018.04.003 29776784 10.1016/j.amepre.2018.04.003 PMC6020687

[CR27] Whitney, C., Evered, J., Patrick, B., & Lee, G. (2024). Whose Um voice is it anyway?? Leveraging Thick transcription to promote inclusion in qualitative research through transcript alignment. *International Journal of Qualitative Methods*, *23*. 10.1177/16094069241256548

